# Terahertz spatial sampling with subwavelength accuracy

**DOI:** 10.1038/s41377-019-0185-3

**Published:** 2019-08-14

**Authors:** Yan Peng, Yiming Zhu, Min Gu, Songlin Zhuang

**Affiliations:** Terahertz Technology Innovation Research Institute, Shanghai Key Lab of Modern Optical System, Terahertz Science Cooperative Innovation Center, University of Shanghai for Science and Technology, 200093 Shanghai, People’s Republic of China

**Keywords:** Applied optics, Optical techniques

## Abstract

A simple terahertz (THz) spatial sampling method offers kilohertz (kHz) level sampling rates and greatly preserves the energy of a THz pulse, which enables THz imaging detection with a high signal-to-noise ratio, micron-grade accuracy, and subwavelength resolution.

To realize real-time and high-fidelity terahertz (THz) images for applications in THz biodetection and communications, we need to reconstruct the 2D profile of a THz beam with high spatial resolution and accuracy. Over the past several years, THz spatial light modulators (THz-SLM) have enabled quasi-real-time THz imaging with frame rates as fast as one image per 2 s (1/2Hz)^1^. This method realized single pixel multiplex THz imaging using an optically controlled reconfigurable THz mask in high-resistivity silicon.

Alternatively, a variety of metamaterial and semiconductor modulation systems have been developed^2–4^. By exciting free photons in metamaterial or semiconductors, the spatially transmitted THz beam can be effectively modulated.

However, the requirements for high-power THz radiation, sample fabrication, and laser excitation increase the complexity of THz spatial modulation systems. In a recent publication, X.-C. Zhang, R. W. Boyd, and coworkers developed a novel near infrared (NIR)-SLM-based THz spatial sampling method that uses only a normal THz irradiation source while achieving up to kHz level sampling rates and micron-grade accuracy^5^. This method can greatly improve the imaging velocity and imaging quality of THz beams, which is beneficial for the widespread use of THz technology in biomedical and industrial sensing applications.

The new design is schematically shown in Fig. 1. The entire system mainly consists of a THz beam, an ‘unknown’ object, a spatially encoded NIR beam, a ZnTe detection crystal and a computational algorithm. Compared to previous reports, this novel THz spatial sampling system has several advances. First, the spatial mask is encoded by using an SLM on the NIR probe beam, which can maximally preserve the energy of the THz pulse and therefore improve the signal-to-noise-ratio (SNR) of the imaging system. Second, this method achieves micron-grade accuracy and subwavelength resolution. For a THz wavelength of 940 μm, the resolution is ~λ/15. Third, this THz spatial sampling method can be combined with different algorithms, which can easily adjust between image quality and reconstruction speed. Finally, a thinner electro-optic (EO) detection crystal with a larger nonlinearity and an SLM with a high switching speed can provide better sampling results. These advantages support many important applications, including THz cameras, biomedical sensing, industrial flaw detection, and security inspection.

**Fig. 1:**
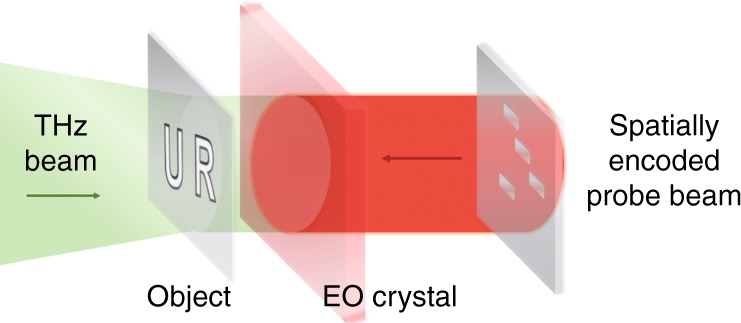
Terahertz spatial sampling imaging system. The THz beam passes through an ‘unknown’ object and irradiates from the left surface of the ZnTe detection crystal. The NIR probe beam travels through the SLM and then propagates to the other side of the ZnTe detection crystal carrying the spatial encoded patterns. The probe pattern spatially overlaps with the THz field and temporally overlaps with the peak position of the THz pulse
